# DNA methylation by three Type I restriction modification systems of *Escherichia coli* does not influence gene regulation of the host bacterium

**DOI:** 10.1093/nar/gkab530

**Published:** 2021-06-28

**Authors:** Kurosh S Mehershahi, Swaine L Chen

**Affiliations:** NUHS Infectious Diseases Translational Research Programme, Department of Medicine, Division of Infectious Diseases, Yong Loo Lin School of Medicine, Singapore 119228; NUHS Infectious Diseases Translational Research Programme, Department of Medicine, Division of Infectious Diseases, Yong Loo Lin School of Medicine, Singapore 119228; Laboratory of Bacterial Genomics, Genome Institute of Singapore, Singapore 138672

## Abstract

DNA methylation is a common epigenetic mark that influences transcriptional regulation, and therefore cellular phenotype, across all domains of life. In particular, both orphan methyltransferases and those from phasevariable restriction modification systems (RMSs) have been co-opted to regulate virulence epigenetically in many bacteria. We now show that three distinct non-phasevariable Type I RMSs in *Escherichia coli* have no measurable impact on gene expression, *in vivo* virulence, or any of 1190 *in vitro* growth phenotypes. We demonstrated this using both Type I RMS knockout mutants as well as heterologous installation of Type I RMSs into two *E. coli* strains. These data provide three clear and currently rare examples of restriction modification systems that have no impact on their host organism’s gene regulation. This leads to the possibility that other such nonregulatory methylation systems may exist, broadening our view of the potential role that RMSs may play in bacterial evolution.

## INTRODUCTION

Regulation and response are fundamental aspects of cellular life. Epigenetics in the context of regulation refers to heritable changes in gene expression without alterations to the primary DNA sequence of an organism (1). This rapidly growing field encompasses an expanding list of biochemical incarnations spanning the entire central dogma (DNA and RNA modifications; chromatin remodelling; noncoding RNAs; and prion proteins) that adds additional layers of complexity and regulatory potential on top of primary DNA sequences (2–5). Epigenetics thus allows a clonal population to generate phenotypic heterogeneity, which can serve specific biological functions (6,7). Particularly for pathogens (and especially facultative pathogens), epigenetics is one way to implement rapid responses to new environments such as different host niches and immune pressures, which in turn may enhance survival and virulence (8,9).

DNA methylation represents the most extensively studied epigenetic mechanism in all domains of life (2,10–12). In eukaryotes, DNA methylation has been demonstrated to play an important role in differentiation, development, and disease (including cancer) (13,14). Bacterial DNA methylation, in particular, has a special place in the collection of epigenetic implementations, as its role ‘outside genetics’ in mediating phage resistance was discovered and characterized prior to the modern usage of the term epigenetics (15).

Bacterial DNA methylation occurs most commonly on adenine (N6-methyladenine, 6mA), but also commonly on cytosine (C5-methylcytosine, 5mC and N4-methylcytosine, 4mC) nucleotides (16). The majority of known bacterial DNA methyltransferases belong to two general categories, restriction modification system (RMS)-associated and orphan methyltransferases (4). RMSs have been characterized as bacterial innate immune systems, using methylation of specific motifs to differentiate between self and non-self DNA (15). RMSs can be found in 90% of all sequenced prokaryotic genomes, with 80% possessing multiple systems, hinting at a diverse and pervasive reservoir of potential epigenetic information (11). RMSs are classified based on co-factor requirement, subunit composition, cleavage pattern, and mechanism into four types (I to IV) (17–20). Type I RMSs were the first discovered and are multi-subunit enzyme complexes consisting of three proteins: a methyltransferase (typically denoted HsdM), an endonuclease (HsdR), and a sequence recognition protein (HsdS). Type I RMSs recognise bipartite sequences (for example, 5′-AAC(N_6_)GTGC-3′, where N = A, T, G or C), with each HsdS subunit possessing two highly variable target recognition domains (TRDs) that specify the two halves of the bipartite motif (19).

Beyond immunity, RMS-associated methyltransferases are also known to regulate gene expression; outstanding examples of this include phasevariable Type I and Type III RMSs. Phase variation of these RMSs results in rapid and reversible changes to methylation patterns, resulting in coordinated changes to the expression of distinct sets of genes (regulons), hence the term ‘phasevariable regulons’ or phasevarions (8,21). Phasevariable RMSs can be identified by known markers of phase variation, such as simple sequence repeats (SSRs) within promoters or gene bodies and *hsdS* genes flanked by inverted repeats. Systematic surveys have identified 13.8% Type I and 17.4% Type III RMSs as being potentially phasevariable (22–24). Several clinically relevant pathogens such as *Helicobacter pylori* (25,26), *Neisseria meningitidis* (27), *Neisseria gonorrhoeae* (28,29), *Haemophilus influenzae* (30,31), *Moraxella catarrhalis* (32,33) and *Kingella kingae* (34) harbor such phasevariable RMSs. An extreme example is found in *Streptococcus pneumoniae*, where some strains carry a phasevariable Type I RMS which can result in up to six distinct methylation specificities, with different methylation patterns specifically associated with either invasive disease or nasopharyngeal carriage *in vivo* (35–37).

In contrast, orphan methyltransferases, as their name suggests, are methylating enzymes without a functional cognate endonuclease. Unlike RMSs, orphan methyltransferases are phylogenetically more conserved across genera/families (11,38,39) and even essential in certain bacteria (40–42). Orphan methyltransferases are not involved in host defense; instead, they play key housekeeping regulatory roles, ranging from DNA replication, mismatch repair, controlling transposition, and gene expression (43). Regarding gene expression, mutation of these orphan methyltransferases can have large effects on the transcriptome, affecting hundreds to thousands of genes (44–49); not surprisingly, therefore, mutation of orphan methyltransferases can also lead to defects in virulence (50–55). In some cases, there exist locus-specific roles of methylation in virulence factor regulation, such as for P pili and the autotransporter antigen 43 (Agn43) (56,57). The two most well-studied orphan methyltransferases are Dam (DNA adenine methyltransferase) in gamma-proteobacteria and CcrM (cell cycle regulated DNA methyltransferase) in alpha-proteobacteria (58).

Therefore, DNA methylation can generally affect both gene expression and phenotypes, of which virulence is an especially interesting case in pathogens. Interestingly, there has been a relative dearth of such studies on the archetypal Type I RMSs, which typically are non-phasevariable and retain both restriction and modification functions. We initially observed that in *Escherichia coli* UTI89, removal of the Type I RMS had no measurable effect on gene expression or virulence, despite >700 DNA bases changing in methylation state. Interestingly, we also found no change in gene expression (i.e. precisely zero significantly differentially expressed genes) when removing the archetypal Type I RMSs from two other *E. coli* strains, MG1655 and CFT073. Intrigued by these findings, we replaced the native UTI89 Type I RMS with the Type I RMS from MG1655 and CFT073; in both cases, installing methylation at ∼400–600 non-native sites in the genome had nearly no effect on gene expression and precisely no effect on a broad panel of bacterial growth phenotypes. These data suggest that, at least in these strains and possibly other *E. coli*, the canonical Type I RMSs are purely host defense mechanisms devoid of any secondary regulatory functions in host physiology and virulence. This is the first reported example of lack of regulation for any native DNA methylation system in bacteria, at least under these *in vitro* conditions (for gene expression) and under >1000 growth conditions (for phenotype).

## MATERIALS AND METHODS

### Ethics statement

All animal experiments were approved by and performed in strict accordance with the Institutional Animal Care and Use Committee (IACUC) of A*STAR (Protocols #110605 and #130853).

### Media and culture conditions

All strains were propagated in Lysogeny Broth (LB) at 37°C unless otherwise noted. Media was supplemented with ampicillin (100 μg*/*ml), kanamycin (50 μg*/*ml), or chloramphenicol (20 μg*/*ml) where required (all antibiotics from Sigma, Singapore). M9 minimal medium (1× M9 salts, 2 mM magnesium sulphate, 0.1 mM calcium chloride, 0.2% glucose) and yeast extract–casamino acids (YESCA) medium (10 g/l casamino acids, 1 g/l yeast extract) were used for specific experiments.

### Strain and plasmid generation

Deletion mutants were generated using a λ-Red recombinase mediated strategy optimised for clinical strains of *E. coli*, with minor modifications (59). Briefly, a positive selection cassette encoding either kanamycin (*neo*) or chloramphenicol (*cat*) resistance was amplified from plasmid pKD4 or pKD3, respectively (60). Primers incorporated, at their 5′-end, 50 bp of homology to the genomic locus being knocked out. The resultant PCR product was transformed into cells expressing λ-Red recombinase from vector pKM208, recovered at 37°C for 2 h with shaking followed by 2 h without shaking, then plated onto the appropriate antibiotic at 37°C overnight.

Strains containing a seamless marker-free replacement of a gene with a different allele were generated using a previously described negative selection strategy (61). Two successive rounds of λ-red recombinase mediated homologous recombination were performed, using the protocol described above, to first replace the wild type allele with a dual positive-negative selection cassette (amplified from plasmids pSLC-217 or pSLC-246 instead of pKD4 or pKD3). A second round of recombination was then performed using a PCR product with the desired allele instead of a resistance cassette, and the second selection done on M9 supplemented with 0.2% rhamnose. Correct clones were confirmed by PCR and by sequencing the recombination junctions by Sanger sequencing (1st Base, Singapore).

The Type I RMS recognition motifs for UTI89 (5′-CCA(N_7_)CTTC-3′), MG1655 (5′-AAC(N_6_)GTGC-3′) or CFT073 (5′-GAG(N_7_)GTCA-3′) and the NotI restriction enzyme site were incorporated into the 5′ end of primers designed to amplify plasmid pACYC184 (which does not natively possess any of these Type I recognition motifs) using an inverse PCR strategy. The resulting linear plasmid amplicons were then digested using NotI, recircularized with T4 DNA ligase, and then transformed into *E. coli* TOP10 to obtain plasmids with one copy of each Type I RMS motif. To generate plasmids with two copies of each Type I motif, the above plasmids served as inverse PCR templates with primers bearing the desired Type I site and a XhoI restriction enzyme site to allow recircularization. The two sites were placed 1500 bp apart (pACYC184 coordinates 381 and 1974). All strains, plasmids and primers (Sigma, Singapore) used in this study are listed in Supplementary Table S1.

### Transformation efficiency assay

Competent cells were prepared using log phase cultures obtained by sub-culturing overnight bacterial culture 1:100 in LB and growing at 37°C up to OD_600_ = 0.4 – 0.5. Cells were washed twice with sterile water followed by a final wash with sterile 10% glycerol and resuspension in 1/100 of the original culture volume of 10% glycerol; these were then stored at –80°C in 50 μl aliquots. Plasmids with one or two copies of the bipartite Type I motif were extracted from the appropriate wild type or Type I RMS mutants to obtain methylated or unmethylated preparations, respectively. Plasmids were extracted using the Hybrid-Q plasmid miniprep kit (GeneAll, South Korea) according to the manufacturer's recommended protocol. Competent cells were transformed with 100 ng of each plasmid using 1mm electroporation cuvettes in a GenePulser XCELL system, at 400 Ω resistance, 25 μF capacitance, and 1700 V output voltage (Bio-Rad, Singapore). Cells were recovered in 1 ml of prewarmed LB at 37°C for 1 h with shaking and plated on selective (chloramphenicol) and non-selective (LB) plates. Transformation efficiency was calculated by dividing the cfu/ml obtained on selective by that on non-selective plates per unit amount of plasmid DNA.

### Mouse infections

*In vivo* infections were performed using a murine transurethral model of urinary tract infection (62). Briefly, bacterial strains were grown in Type I pili-inducing conditions by two passages in LB broth at 37°C for 24 h without shaking; a 1:1000 dilution was made from the first to the second passage. Cells were then harvested by centrifugation and resuspended in sterile cold PBS to OD_600_ = 1. Type I piliation for each strain was evaluated by a hemagglutination assay and Type I phase assay as described previously (63). A 1:2 dilution of the PBS suspension (final OD_600_ = 0.5) was then used as the inoculum. 7–8 week old female C3H/HeN mice (InVivos, Singapore) were anaesthetized using isoflurane and 50 μl of inoculum (OD_600_ = 0.5, ∼1–2 × 10^7^ cfu/50 μl) was transurethrally instilled into the bladder using a syringe fitted with a 30 gauge needle covered with a polyethylene catheter (Product #427401, Thermo fisher scientific, USA). At specified times, mice were sacrificed and bladders and kidneys were harvested aseptically and homogenized in 1 ml and 0.8 ml of sterile PBS, respectively. Ten-fold serial dilutions were plated on appropriate selective plates to quantify bacterial loads. For co-infections, the inoculum consisted of a 1:1 mixture of two strains with an antibiotic resistance cassette inserted at the phage HK022 attachment site *attP* (64), each at 1–2 × 10^7^ CFU/50 μl; otherwise, the procedure was identical to that described for the single infections above. To account for potential bias due to selection markers, co-infections were performed with an equal number of mice infected with strains with their selection marker combinations reversed. For example, co-infections comparing wild type (Kan^R^) and mutant (Chlor^R^) were also performed with wild type (Chlor^R^) and mutant (Kan^R^). Bacterial titres from each organ and starting inoculum were used to calculate the competitive index (CI) as follows: CI = (output wild type/output mutant)/(input wild type/input mutant).

### RNA sequencing

Stationary phase cells were obtained by propagating strains statically for two serial 24 h passages at 37°C, with a 1:1000 dilution between passages (identical to the inoculum used for mouse infections). Log phase cells were obtained by diluting overnight cultures 1:100 in LB and growing to log phase (OD_600_ = 0.4–0.5) at 37°C with shaking. RNA was extracted with the RNeasy Mini kit (Qiagen, Singapore) from three biological replicates for both log and stationary phase samples using 7 × 10^8^ cells. RNA quality was assessed using the Agilent RNA 6000 pico kit on an Agilent 2100 Bioanalyzer (Agilent Technologies, USA); only replicates where all samples had an RNA integrity number (RIN) ≥7 were used. Ribosomal RNA (rRNA) depletion was done with the Ribo-Zero rRNA removal kit (Epicenter, USA) according to the manufacturer's recommended protocol. Libraries were generated using the ScriptSeq v2 RNA-seq library preparation kit (Epicenter, USA) according to the manufacturer's recommended protocol. Each uniquely indexed strand specific library was assessed for library size and amount using the Agilent DNA 1000 kit (Agilent Technologies, USA). After normalization and pooling, samples were sequenced on either the Illumina HiSeq 4000 or NextSeq sequencer with 2 × 151 bp or 2 × 76 bp reads (Illumina, USA).

Raw sequencing reads were mapped to their respective reference genomes: RefSeq accession GCF_000013265.1 for *E. coli* UTI89, GCF_000005845.2 for *E. coli* MG1655 and GCF_000007445.1 for *E. coli* CFT073 using BWA-MEM (version 0.7.10) with default parameters (65). HTseq was used to quantify sequencing reads mapping to predicted open reading frames (ORFs) (66). Ribosomal RNA (rRNA) and transfer RNA (tRNA) sequences (based on the corresponding Genbank RefSeq annotation) were filtered out of the data set. R (version 2.15.1) was used for differential expression analysis, using the edgeR package (67). Briefly, samples were normalized by TMM (trimmed median of means), common and tagwise dispersion factors were estimated using a negative binomial model, and then fold change values were calculated from these normalized counts. A log_10_ false discovery rate (FDR) cutoff of ≤0.05 and a log_2_ fold change ≥1.5 were applied, resulting in the final set of differentially expressed genes. RNA sequencing quality metrics are listed in Supplementary Table S2.

### Pacific Biosciences single molecule real time (SMRT) sequencing

Genomic DNA was extracted from log phase bacterial cultures grown in LB at 37°C with shaking and quantified using a Qubit 2.0 Fluorometer (Life Technologies, USA) using the dsDNA HS kit. 5 μg of DNA was sheared to 10 kb using a g-Tube (Covaris, USA), and a SMRTbell library was made with the SMRTbell template prep kit 1.0 (Pacific Biosciences, USA) according to the manufacturer's instructions. Library quality and quantity were assessed using the Agilent DNA 12,000 kit (Agilent Technologies, USA) and sequenced on the PacBio RS II sequencer (Pacific Biosciences, USA). Sequencing was performed using a single SMRTCell with P4-C2 enzyme chemistry using a 180 min movie. Reads were mapped back to the corresponding reference genome (as indicated under ‘RNA Sequencing’) and methylated motifs were identified using the ‘RS_Modification_and_motif_analysis’ algorithm in SMRT Analysis suite v2.3 using default parameters. Bases with a coverage of at least 25× and a methylation quality value (QV) of at least 60 (default values for the RS_Modification_and_motif_analysis protocol) were identified as being methylated.

### Methylation site distribution analysis

Transcription start sites (TSSs) in *E. coli* MG1655 have been determined by multiple methods (68–70). We extracted the start site locations (and corresponding direction of transcription) from each of these, which ranged from 3746 (70) up to 16,359 (68) start sites. One report examined *E. coli* grown in LB at OD 0.4 and 2.0 (69); for these, we analyzed the conditions independently (i.e. only those start sites detected in each growth condition). These publications used the NC_000913.2 (69,70) or U00096.2 (68) (Genbank accessions) reference sequences, which are identical. We took a 50 bp window upstream of each TSS (upstream defined relative to the direction of transcription) as putative promoters and searched for overlaps with the location of EcoKI, EcoUTI89I, EcoCFTI and Dam methylation sites. To assess whether there was significant enrichment or depletion of these sites within these putative promoter sequences, we performed a bootstrapping analysis with 10,000 replicates. For each replicate, a random number (between 1 and the length of the genome) was chosen and added to all the TSS positions; then 50 bp windows upstream of the new positions (modulo the length of the genome) were searched for the same methylation sites. The bootstrap *P*-value is the number of replicates with equal or fewer methylation sites for each motif. A Bonferroni correction was applied prior to determining statistical significance (*P* < 0.05).

### Quantitative RT-PCR

Samples were prepared as described under ‘RNA sequencing’, using the RNeasy Mini kit (Qiagen, Singapore). 1 μg of RNA was treated with DNase I, RNase-free (Thermo fisher scientific, USA) at 37°C for 1 h to remove any residual genomic DNA. Next, 500 ng of RNA was used for cDNA synthesis with SuperScript II Reverse Transcriptase and Random hexamers (Invitrogen, USA) according to the manufacturer's recommended protocol. Amplified cDNA was diluted 1:4 for all target genes and 1:400 for the *rrsA* internal control. Real-time PCR was performed with the KAPA SYBR FAST qPCR kit (KAPA Biosystems, USA) on a LightCycler 480 instrument (Roche, Singapore) with the following cycle: 95°C for 5 min, followed by 40 cycles of 95°C for 30 s and 60°C for 30 s. Target-specific primers for qRT-PCR were designed using the IDT PrimerQuest tool (IDT, Singapore) and are listed in Supplementary Table S1. Relative fold change for target genes was calculated by the ΔΔCT method utilizing the 16S ribosomal gene *rrsA* as the internal control. Reverse transcriptase and negative controls were included in each run.

### Motility assay

Motility assays were performed as described previously with slight modifications (71). Strains were grown to log phase (OD_600_ = 0.4–0.5) by sub-culturing overnight bacterial cultures 1:100 in LB at 37°C. Bacteria were harvested by centrifugation (3200 g, 10 min) and resuspended in sterile PBS to OD_600_ = 0.4. Sterile 0.25% LB agar plates were prepared and stabbed once with each strain using sterile toothpicks. The soft agar plate was then incubated at 37°C for 7–8 h. Motility was calculated by measuring the diameter of the bacterial motile front. Distances were normalized to a wild type control included in each experiment.

### Biofilm assay

A 96-well Crystal Violet biofilm assay was performed as previously described (72). Briefly, strains were grown to log phase (OD_600_ = 0.4–0.5) by sub-culturing overnight bacterial culture 1:100 in LB at 37°C and resuspended in sterile PBS to OD_600_ = 0.4. 96-well clear flat bottom poly vinyl chloride (PVC) plates (Product #01816049, Costar, Singapore) were seeded with 200 μl of sterile media (LB or YESCA) and 5 μl of the PBS suspension. PVC plates were incubated for the specified times at 26°C or 37°C in a humidified chamber. Plates were washed once with water and stained with Crystal Violet for 30 min. Excess stain was removed by washing thrice with water. Residual stain was then dissolved in 200 μl of 50% ethanol, with care taken to avoid disturbing the biofilm. The amount of biofilm was quantified by measuring OD at 590 nm using a Sunrise 96 well microplate absorbance reader (Tecan, Switzerland). The biofilm produced by each test strain was normalized to that of the corresponding wild type strain.

### Growth curves

Growth curves for bacterial strains were measured using the Bioscreen C instrument (Bioscreen, Finland). Strains were grown to log phase (OD_600_ = 0.4–0.5) by sub-culturing overnight bacterial culture 1:100 in LB at 37°C and resuspended in sterile PBS to OD_600_ = 0.4. 5 μl of this normalized bacterial suspension was then inoculated into 145 μl of the desired growth media (LB (rich) or M9 (minimal)) in triplicates. Plates were incubated at 37°C and OD_600_ was measured every 15 min for 20 h.

### Biolog phenotype microarray (PM)

PM assays (73) were performed using PM plates 1 to 20 by the PM services group at Biolog, USA using their standard protocol for *E. coli*. Succinate was added as the carbon source for PM plates 3–20 and all experiments with UTI89 (a niacin auxotroph) included 1 μg/ml niacin. All experiments were done with biological duplicates. Briefly, PM plates contain: PM1–2 Carbon sources, PM3 Nitrogen sources, PM4 phosphorous and sulphur sources, PM5 Nutrient supplements, PM6-8 Peptide nitrogen sources, PM9 Osmolytes, PM10 pH and PM11-20 inhibitors for different metabolic pathways. The PM services group analyzed growth curves, as measured colorimetrically, using their own proprietary software. Gain or loss of a phenotype/resistance was called by the PM services group, again using their proprietary software, which identified phenotypic differences based on the presence of a height difference between the strains in both replicates and a quality score >150 as cutoffs.

## RESULTS

### Uropathogenic *Escherichia coli* UTI89 possesses a functional type I restriction modification system

The clinical cystitis strain UTI89 encodes a single putative Type I RMS at the *hsdSMR* locus (74). According to the REBASE database, this RMS belongs to a group of 104 *E. coli* Type I systems, with Eco646I as the prototype system and all of which share the same recognition sequence (5′-CCA(N_7_)CTTC-3′). The REBASE annotations for the genes constituting the UTI89 Type I RMS are S.EcoUTORF5051P, M.EcoUTORF5051P and EcoUTORF5051P (75,76). PacBio single molecule real time (SMRT) sequencing identified three methylation motifs in UTI89: 5′-GATC-3′, 5′-CCWGG-3′ and 5′-CCA(N_7_)CTTC-3′ (Supplementary Table S3). The first two motifs are well known in *E. coli* as the methylation sites of orphan methyltransferases Dam and Dcm, respectively (12,77). The third motif, a bipartite N6-methyladenine (m6A) motif, is typical for a Type I RMS (19). Deletion of the *hsdSMR* locus in UTI89 resulted in loss of adenine methylation only at the 5′-CCA(N_7_)CTTC-3′ motif, confirming the specificity of this RMS (Supplementary Table S3). 97.7–99.3% of all occurrences of this motif were detected as methylated in the wild type strain, which is similar to detection levels for other RMSs considered to be constitutively active (Supplementary Table S3) (11). Furthermore, there are no other homologs of any of the *hsdSMR* genes in the UTI89 genome, and no simple sequence repeats or inverted repeats suggestive of phase variation are identifiable at the *hsdSMR* locus in UTI89.

**Figure 1.**
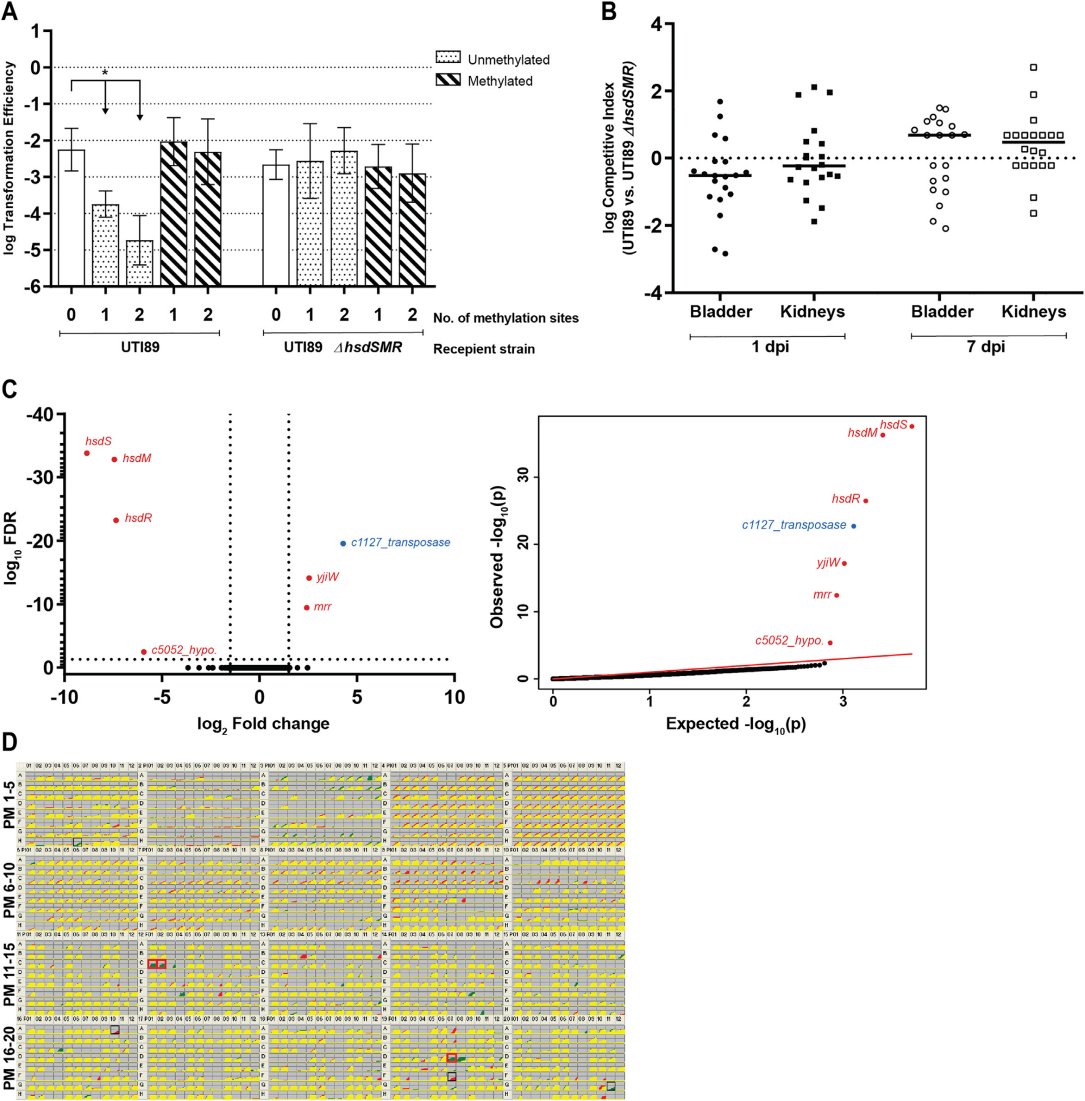
UTI89 Type I RMS EcoUTI89I functions only as a restriction system, with no apparent regulatory role. (**A**) Transformation efficiency assay using plasmids bearing 0, 1 or 2 copies (as indicated on the x-axis) of the UTI89 Type I RMS motif (5′-CCA(N_7_)CTTC-3′). Recipient cells were wild type UTI89 and the isogenic *ΔhsdSMR* mutant, as indicated by the labels below the x-axis. Unmethylated and methylated plasmid preparations were used to transform each strain, as indicated by the legend at the top right. An unpaired t-test was used to identify significant differences between plasmids with 0, 1 and 2 methylation sites for both preparations and strains; * *P* < 0.05, *n* = 3 biological replicates. Data represents mean ± standard deviation (s.d.) of log transformed values. (**B**) Competitive index (CI) for *in vivo* co-infections with wt UTI89 and UTI89*ΔhsdSMR*. Bladder and kidney pairs (as indicated on the x-axis) were aseptically harvested at 1 or 7 days post infection (dpi), as indicated by the labels below the x-axis, and plated on appropriate selective plates for calculation of CI. A Wilcoxon signed rank test was used to test for a significant difference of log CI from 0; * *P* < 0.05, *n* = 20 mice/time point, performed as two biological replicates. Each point represents data from a single mouse; horizontal lines represent the median. (**C**) RNA sequencing comparing logarithmic phase transcriptomes of UTI89 and UTI89*ΔhsdSMR*. Left, a volcano plot of log_10_ FDR against log_2_ fold change. Right, qq-plots showing the distribution of uncorrected *P*-values. Significantly differentially expressed genes (log_2_ fold change >1.5 and log_10_ FDR <0.05) are labelled and colored either red (deleted genes/polar effects) or blue (validated as false positive by qRT-PCR). *n* = 3 biological replicates. (**D**) Phenotype microarray (PM) panel with plates PM1 to 20 comparing wt UTI89 and UTI89*ΔhsdSMR*. Each plate is represented as a 12 × 8 grid of growth curves (red (wt), green (*ΔhsdSMR*), and yellow (overlap) on a gray background). Each growth curve plots growth (measured colorimetrically) (y-axis) against time (x-axis). Wells representing conditions where a height difference was observed between the strains in both replicates are boxed in black, and wells which also have a quality score >150 are considered significant and boxed in red. *n* = 2 biological replicates.

To verify that the UTI89 *hsdSMR* locus was functional for restriction of incoming DNA, we performed a classic plasmid transformation efficiency assay (76). A plasmid containing the 5′-CCA(N_7_)CTTC-3′ motif was isolated from the UTI89*ΔhsdSMR* strain (which does not methylate this motif); this plasmid was transformed less efficiently into wild type (wt) UTI89 than into an otherwise isogenic UTI89*ΔhsdSMR* strain (Figure 1A). Moreover, as seen with other Type I RMSs, addition of a second motif further reduced the efficiency of transformation into wt UTI89 (Type I RMSs cleave DNA when translocation is stalled due to collision between adjacent Type I complexes (78)). When plasmids were isolated from wt UTI89 (which methylates the 5′-CCA(N_7_)CTTC-3′ motif), no difference in efficiency was seen between transformations into UTI89 and UTI89*ΔhsdSMR* (Figure 1A). Thus, uropathogenic *E. coli* UTI89 possesses a functional, archetypal, non-phasevariable Type I RMS (similar to the MG1655 Type I RMS EcoKI) with a formal name of RM.EcoUTI89I and a specificity of 5′-CCA(N_7_)CTTC-3′ (79). We hereafter refer to this system as EcoUTI89I.

### EcoUTI89I Type I methylation has no detectable role during *in vivo* urinary tract infection

DNA methylation is widely recognized to influence gene expression and virulence in multiple bacterial pathogens (51,58,80). EcoUTI89I methylates 754 sites in the UTI89 genome; we therefore hypothesized that complete removal of this system would affect both gene expression and virulence. The UTI89 and UTI89*ΔhsdSMR* strains had no difference in growth in both rich and minimal media nor in Type I pilus expression *in vitro* (data not shown). We then tested virulence using a transurethral murine model for ascending urinary tract infection (UTI) (62). Competitive co-infections in 6–8 week old C3H/HeN mice using equal mixtures of wild type UTI89 and UTI89*ΔhsdSMR* showed no competitive advantage for either strain at 1 or 7 days post-infection (dpi) in either bladders or kidneys (Figure 1B). Comparison of bacterial loads from single infections also showed no significant difference between the two strains (Supplementary Figure S1).

### EcoUTI89I Type I methylation has no effect on gene expression or growth in multiple conditions

The lack of an *in vivo* infection phenotype, despite the complete removal of EcoUTI89I methylation, contrasts with reports on multiple other methylation systems (including RMSs) that impact bacterial phenotypes (47,81–85). We therefore asked whether changing methylation could alter any gene expression using RNA-seq. When comparing UTI89 with the otherwise isogenic UTI89*ΔhsdSMR* strain, in both log and stationary phase in rich media, only seven genes were significantly changed in expression in the mutant: the (deleted) *hsdSMR* and UTI89_C5052 genes were downregulated, the two genes flanking the *hsdSMR* locus were upregulated, and a single transposase gene was upregulated. In the UTI89 genome, UTI89_C5052 is a hypothetical gene annotated in the intergenic region between *hsdM* and *hsdR*, so it was deleted with the entire *hsdSMR* locus, accounting for it being called as downregulated. Altered expression of the two flanking genes was connected to the use of an antibiotic resistance cassette to knock out *hsdSMR* (see methods). Targeted qRT-PCR on the transposase gene was unable to validate the change seen in RNA-seq (Figure 1C, Supplementary Figure S2A, Supplementary Tables S4 and S5). Therefore, we could find no change in gene expression that was attributable to loss of Type I methylation.

Although RNA-seq is a powerful tool for assaying gene expression, it only tests expression in a limited number of experimental growth conditions. To test a broader range of conditions, we used Biolog Phenotype Microarrays (PM) (73). PM plates 1–20 represent 1190 different nutrients, toxins, antibiotics, inhibitors and other conditions. Relative to wt UTI89, the UTI89*ΔhsdSMR* strain had gained resistance to the aminoglycoside antibiotic paromomycin (PM12, wells C01-02), which was consistent with the use of a kanamycin resistance cassette to knock out the *hsdSMR* locus. (Figure 1D, Supplementary Table S6). UTI89*ΔhsdSMR* had also gained resistance to the formazan dye Iodonitrotetrazolium (INT) violet (PM19, well D07). There are four wells containing different concentrations of INT on PM19 (D05 - D08); well D07 is the second-lowest concentration, and the other three wells were not called as significantly different from wt UTI89. In particular, at the two highest concentrations (wells D05 and D06) there was very little difference in the growth curves between UTI89 and UTI89*ΔhsdSMR*. We therefore suspect that this was a false positive phenotype (Figure 1D, Supplementary Table S6). We thus find, strikingly, no phenotypic difference attributable to the loss of methylation.

### Loss of Type I methylation in two other *E. coli* strains also has no effect on gene expression or growth phenotypes

**Figure 2.**
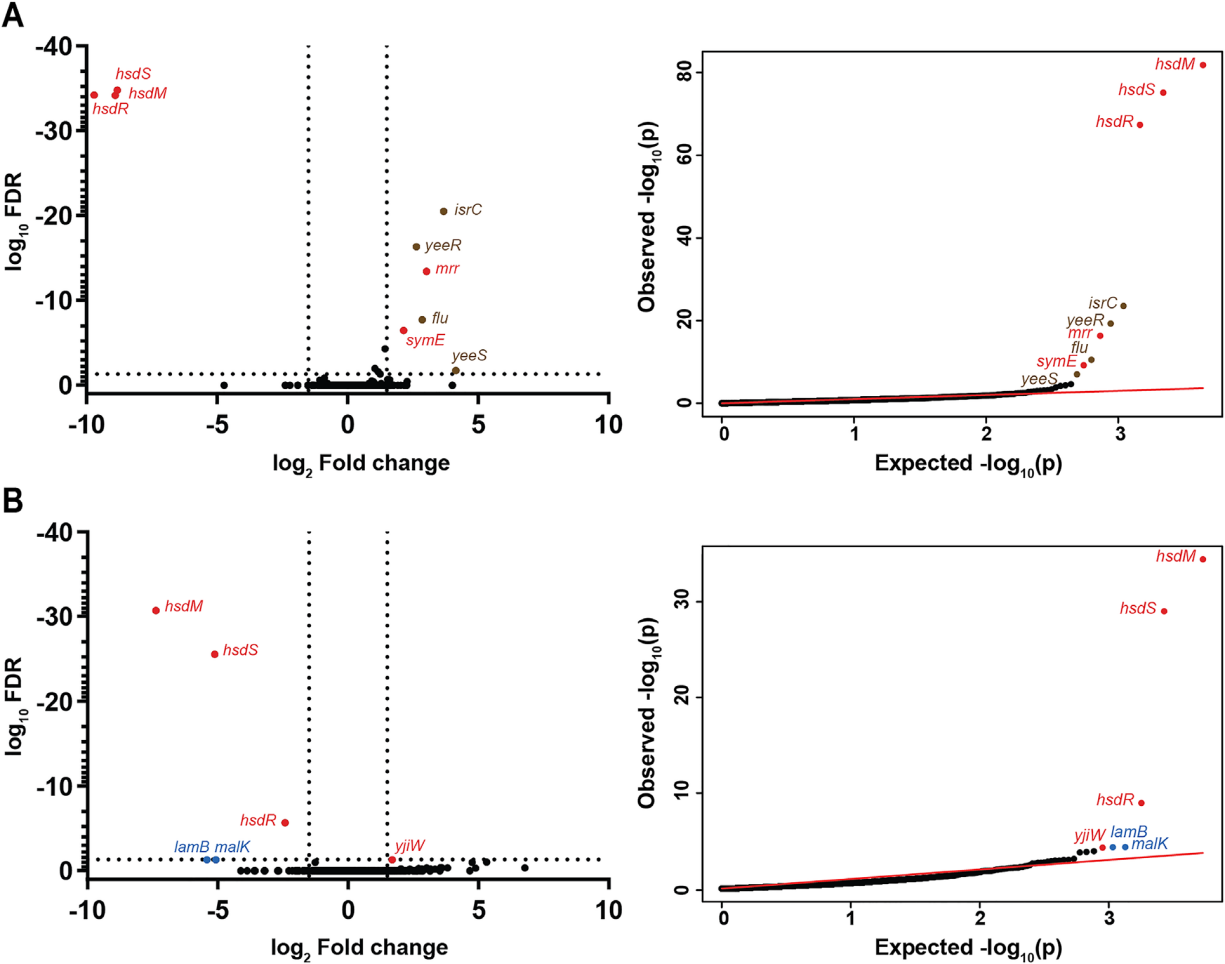
Type I RMSs from *E. coli* CFT073 and MG1655 do not affect gene expression. RNA sequencing comparing logarithmic phase transcriptomes of (**A**) MG1655 and MG1655*ΔhsdSMR*, and (**B**) CFT073 and CFT073*ΔhsdSMR*. Left, a volcano plot of log_10_ FDR against log_2_ fold change. Right, qq-plots showing the distribution of uncorrected *P*-values. Significantly differentially expressed genes (log_2_ fold change >1.5 and log_10_ FDR <0.05) are labelled and colored either red (deleted genes/polar effects), brown (differentially expressed genes) or blue (validated as false positive by qRT-PCR). *n* = 2 (**A**, MG1655) or 3 (**B**, CFT073) biological replicates.

We next asked whether the lack of any detectable gene expression or growth phenotype changes was unique to UTI89 and the EcoUTI89I methylation system. *E. coli* strain MG1655 is a well-studied, lab-adapted K12 strain, while CFT073 is a commonly used pyelonephritis strain; both also carry a single non-phasevariable Type I RMS (EcoKI and EcoCFTI, respectively) similar to EcoUTI89I. EcoKI methylates the 5′-AAC(N_6_)GTGC-3′ motif, while EcoCFTI is predicted to methylate the 5′-GAG(N_7_)GTCA-3′ motif (75,86). Plasmid transformation efficiency assays confirmed that both EcoKI and EcoCFTI also were functional as restriction systems for incoming DNA, targeting the previously reported motifs (Supplementary Figure S3). In RNA-seq experiments, besides the expected change in expression of the *hsdSMR* genes themselves and the flanking genes affected by insertion of the antibiotic resistance cassette, MG1655*ΔhsdSMR* had 4 genes upregulated during log phase, all encoded within a predicted cryptic prophage and validated by qRT-PCR (Figure 2A, Supplementary Figure S2B, Supplementary Tables S4 and S5). However, independently generated clones of the MG1655*ΔhsdSMR* strain did not have a similar upregulation of these four prophage genes when tested by qRT-PCR; we therefore consider the expression changes in these four genes an artefact of that single strain, possibly due to a second site mutation during generation of the knockout (data not shown). The CFT073*ΔhsdSMR* strain had limited changes in gene expression relative to the wild type CFT073, including the *hsdSMR* genes, and flanking gene *yjiW* (polar effect). Targeted qRT-PCR on *malK* and *lamB* genes was unable to validate the changes seen in RNA-seq, confirming these as false positives (Figure 2B, Supplementary Figure S2C, Supplementary Tables S4 and S5).

Using the Biolog PM, we again found no phenotypic differences between either (i) MG1655 and MG1655*ΔhsdSMR* or (ii) CFT073 and CFT073*ΔhsdSMR*; except for gained antibiotic resistance due to kanamycin resistance cassette used to knock out the *hsdSMR* locus (Supplementary Table S6, Figure S4A and S4B). Thus, in three distinct *E. coli* strains, each encoding Type I RMSs with different specificities, deletion of the entire RMS had no detectable effect on gene expression or any growth phenotypes we measured.

### Switching Type I methylation systems in UTI89 also does not affect gene expression or any growth phenotypes

**Figure 3.**
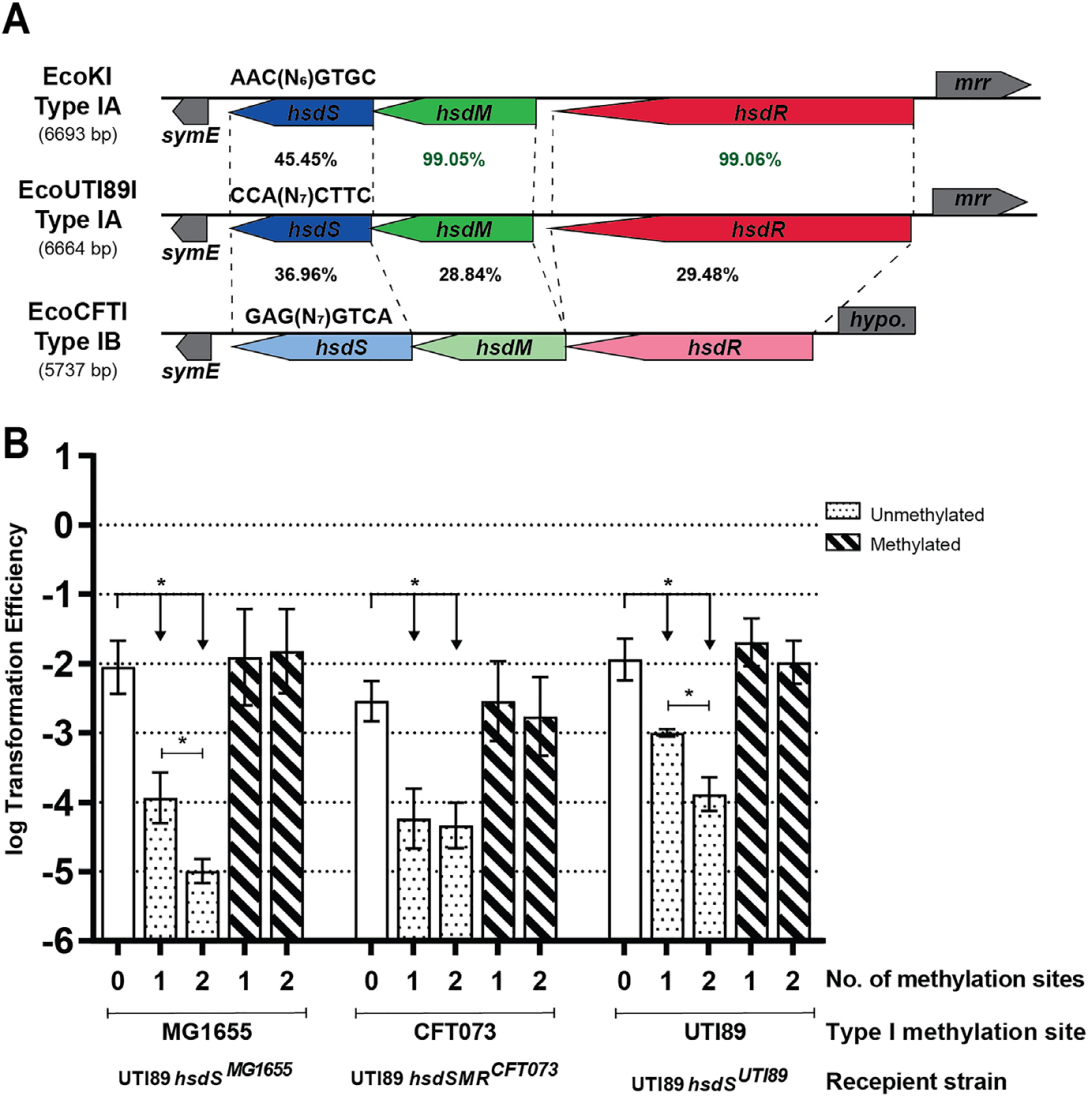
Installation of different Type I methylation specificities into UTI89. (**A**) Graphical representation of the host specificity determinant (*hsd*) locus showing the amino acid identity between Type I RMSs EcoKI, EcoUTI89I, and EcoCFTI from *E. coli* strains MG1655, UTI89, and CFT073, respectively. The subclassification for each system is indicated on the left, with the total length of DNA spanned by the *hsd* genes shown in parentheses. The *hsd* genes are shown in different colors for visualization and drawn to scale, with transcription direction indicated by the arrow. Dotted lines further connect the corresponding beginnings and ends of each *hsd* gene in different systems. Genes adjacent to the *hsd* genes are shown in gray. The percentage identity between *hsd* genes from different systems is indicated; green >99% and black <50%. (**B**) Transformation efficiency assay using plasmids bearing 0, 1 or 2 copies (as indicated on the x-axis) of the MG1655 (5′-AAC(N_6_)GTGC-3′), CFT073 (5′-GAG(N_7_)GTCA-3′) and UTI89 (5′-CCA(N_7_)CTTC-3′) Type I RMS motifs. Recipient cells were UTI89 *hsdS^MG1655^*, UTI89 *hsdSMR^CFT073^* and UTI89 *hsdS^UTI89^*, as indicated by the labels below the x-axis. Unmethylated and methylated plasmid preparations were used to transform each strain, as indicated by the legend at the top right. An unpaired t-test was used to identify significant differences between plasmids with 0, 1 and 2 methylation sites for both preparations and strains; * *P* < 0.05, *n* = 3 biological replicates. Data represents mean ± s.d. of log transformed values.

Type I RMSs can be highly polymorphic among different strains of the same species. In many cases, this is thought to be due to both mutation and recombination, possibly driven by diversifying selection (87,88). Furthermore, the whole *hsdSMR* locus need not be recombined; Type I RMSs are classified into five families (designated A through E) based on the similarity of the *hsdM* and *hsdR* genes, enabling intra-family genetic complementation with divergent *hsdS* genes (19). As *hsdS* encodes the specificity determinant, mutation or recombination of just the *hsdS* gene is sufficient to alter the methylation and restriction specificity of the entire system (35,89). The RMSs in UTI89 and MG1655 are both subclassified as Type IA and demonstrate this latter relationship; the *hsdM* and *hsdR* genes in these two strains have very similar sequences (99% identical), while the *hsdS* genes are only 45.4% identical and direct distinct specificities (Figure 3A). The Type I RMS in CFT073, on the other hand, is a Type IB RMS with <40% identity to the Type IA system in all three genes (Figure 3A).

To analyze the effect of changing Type I methylation specificity, we created two derivatives of UTI89: one where the *hsdS* gene was replaced by the MG1655 *hsdS* allele (UTI89 *hsdS^MG1655^*), and one where the entire *hsdSMR* locus was replaced by the EcoCFTI locus (UTI89 *hsdSMR^CFT073^*). As a control, we also re-inserted the UTI89 *hsdS* allele (UTI89 *hsdS^UTI89^*) using the same cloning strategy as that used to make UTI89 *hsdS^MG1655^* (i.e. UTI89 *hsdS^MG1655^* and UTI89 *hsdS^UTI89^* share the same parental strains and have undergone the same cloning steps). PacBio SMRT sequencing confirmed that in plasmids isolated from each of these strains only the expected motif was methylated (data not shown). Plasmid transformation efficiency assays also showed that all 3 ‘methylation-switch’ strains generated indeed had functional Type I RMSs with specificities as expected based on the encoded *hsdS* allele (i.e. UTI89 *hsdS^MG1655^*, UTI89 *hsdSMR^CFT073^*, and UTI89 *hsdS^UTI89^* restricted only plasmids carrying the EcoKI, EcoCFTI, and EcoUTI89I motif respectively, in a methylation-dependent manner) (Figure 3B).

**Figure 4.**
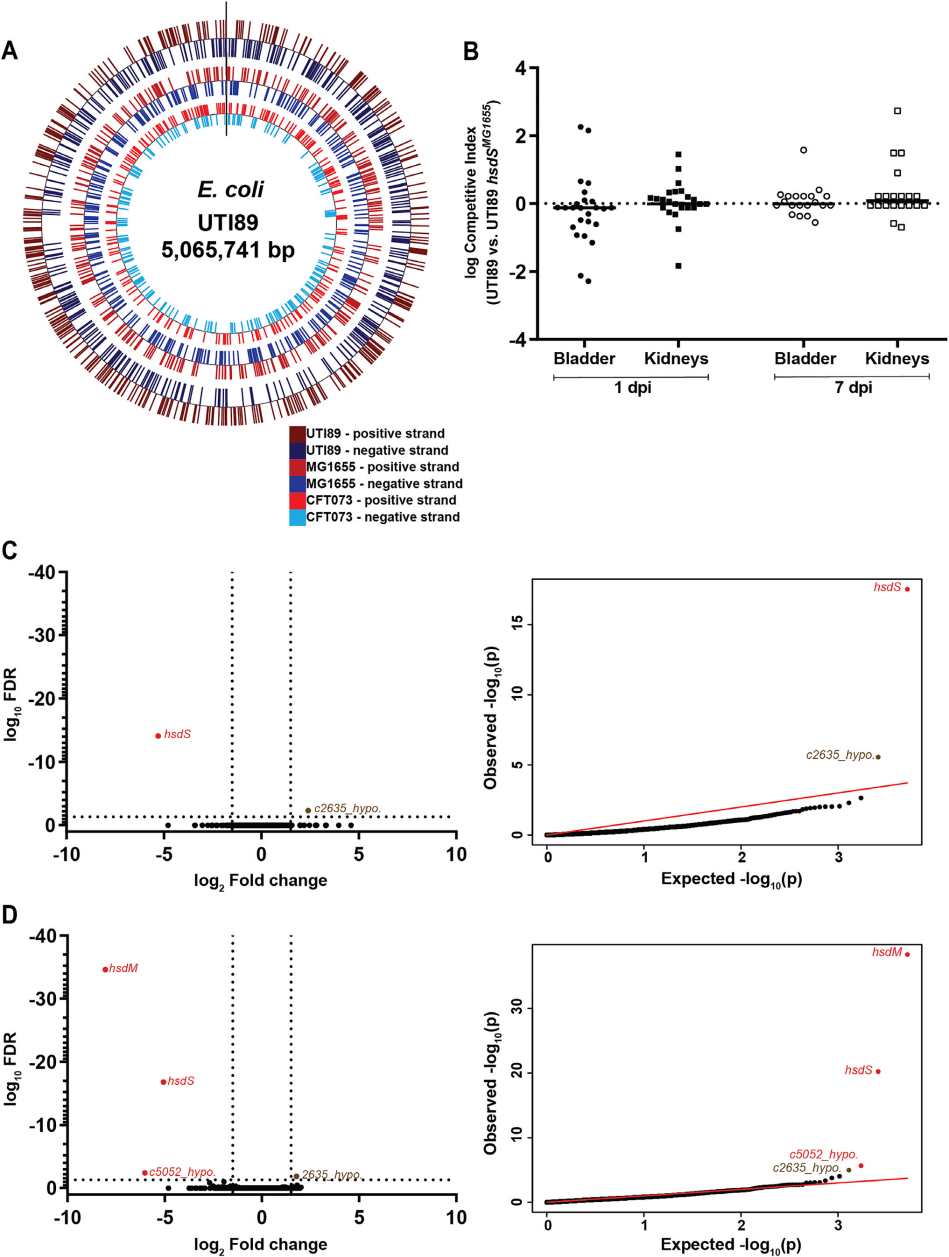
Heterologous Type I methylation in UTI89 does not affect virulence and gene expression. (**A**) Distribution of heterologous Type I methylation sites in the UTI89 genome. Methylation sites for different systems are indicated by tick marks on different concentric circles; from the outer to the inner circle, UTI89 (747 sites), MG1655 (628 sites), and CFT073 (415 sites) sites are represented. Positions on the positive and negative strands are represented by colored tick marks as indicated in the legend at the bottom right, marked in red and blue respectively. (**B**) Competitive index (CI) for *in vivo* co-infections with wt UTI89 and UTI89 *hsdS^MG1655^*. Bladder and kidney pairs (as indicated on the x-axis) were aseptically harvested at 1 or 7 days post infection (dpi), as indicated by the labels below the x-axis, and plated on appropriate selective plates for calculation of CI. A Wilcoxon signed rank test was used to test for a significant difference of log CI from 0; * *P* < 0.05, *n* = 25 mice (1dpi) and 20 mice (7dpi), performed as two biological replicates. Each point represents data from a single mouse; horizontal lines represent the median. (**C** and **D**) RNA sequencing comparing logarithmic phase transcriptomes of (**C**) UTI89 and UTI89 *hsdS^MG1655^*, and (**D**) UTI89 and UTI89 *hsdSMR^CFT073^*. Left, a volcano plot of log_10_ FDR against log_2_ fold change. Right, qq-plots showing the distribution of uncorrected *P*-values. Significantly differentially expressed genes (log_2_ fold change >1.5 and log_10_ FDR <0.05) are labelled and colored either red (deleted genes/polar effects) or brown (differentially expressed genes). *n* = 3 biological replicates.

These strains introduce two different Type I methylation specificities in the UTI89 genome, accounting for 641 (MG1655) and 423 (CFT073) predicted newly methylated sites (Figure 4A, Supplementary Table S7). All together, the three RMSs we examined account for 1818 distinct methylation sites that would vary in methylation status among the strains we studied. The number of intergenic methylation sites in UTI89, which are expected to be more likely to affect gene regulation (46,90–93), are comparable for the three Type I RMSs (31–40 intergenic sites). Assuming an unbiased distribution across the genome, each of the three 7 bp Type I recognition motifs are present in the putative promoters (50 bp upstream) of approximately only 0.31% of the open reading frames (ORFs) present in UTI89, MG1655, and CFT073 (Supplementary Table S7). Using RNA-seq, we again found no gene expression changes for any of the ‘methylation-switch’ strains compared with the parental wt UTI89, except for one hypothetical gene that was close to both the FDR and fold-change cutoffs (Figure 4C and D, Supplementary Figure S2D–S2G, Tables S4 and S5). Furthermore, Biolog PM also showed no significant changes attributable to Type I methylation in UTI89 *hsdS^MG1655^* (Supplementary Table S6, Figure S4C and S4D). Notably, there was no difference in INT resistance between UTI89 *hsdS^MG1655^* and wt UTI89 at any concentration, consistent with our previous interpretation that the single difference identified between UTI89 and UTI89*ΔhsdSMR* was a false positive (Figure 1D, Supplementary Table S6).

As a final functional test, we used several *in vitro* and *in vivo* assays for virulence. Changing the methylation system had no effect on growth rate in rich or minimal media or on virulence-related assays such as motility and biofilm formation (Supplementary Figure S5A–S5G). Competitive co-infections with UTI89 and UTI89 *hsdS^MG1655^*again revealed no competitive advantage for either strain, irrespective of the time-point or organ tested (Figure 4B).

To test whether UTI89 was unique in its ability to tolerate changes in methylation, we created a similar ‘methylation-switch’ in MG1655 by inserting the UTI89 *hsdS* allele. We again found no differences between wt MG1655 and MG1655 *hsdS^UTI89^* in the Biolog PM phenotype screen (Supplementary Table S6, Figure S4E and S4F). We therefore conclude that, at least in UTI89 and MG1655, substantial changes in DNA methylation across the genome are not merely tolerated but simply have no measurable effect on gene expression or laboratory-measured phenotypes.

## DISCUSSION

The EcoKI restriction modification system (RMS) was identified over a half century ago due to its role in the specific restriction of exogenous (phage) DNA (86). EcoKI is thus the prototypical example of a RMS, but subsequent studies also demonstrated that, particularly for *E. coli* but also for many other bacteria, this was an archetypal system as well. It was thus designated ‘Type I’ in the RMS classification that followed. As more RMSs were discovered, they were initially assumed to play similar roles in host defense and horizontal gene transfer (94,95). In bacteria, one of the early indications that methylation could affect transcription arose from the study of regulation of RMSs themselves, some of which utilize methylation as a readout of expression level, effectively a type of product-inhibition feedback (96). The general observation that DNA methylation, particularly that mediated by orphan methyltransferases (such as Dam or CcrM, which may themselves have evolved from RMSs), could alter transcription (44,48,97), led to the discovery of a broad suite of associated roles in DNA metabolism, cell physiology, and virulence (12,51,55,98–100). An active research community continues to describe and characterize the additional roles that DNA methylation (from both restriction and orphan systems) play in bacteria (4,39,101), some of which can be very specific (81,83). We now show that, in contrast with expectations from this literature, the archetypal EcoKI Type I RMS in MG1655 has exactly zero impact on gene regulation or any phenotype among >1000 growth conditions tested. These results generalize to two additional Type I RMSs from pathogenic *E. coli* strains UTI89 and CFT073, with removal of Type I methylation having no detectable impact on gene regulation or cellular phenotype.

The paradigm of ‘one gene, one function’ made the initial discovery that restriction systems could play a distinct epigenetic role in gene regulation somewhat surprising. Now, the idea that methylation can affect the interaction with DNA binding proteins is quite clear (12), and orphan methyltransferases were thought to have preserved DNA methylation for such non-defense (i.e. regulatory) functions (11). Perhaps the strongest evidence that gene regulation is a biologically selected function for RMSs comes from the discovery of phasevarions, in which phasevariable expression (through recombination or simple sequence repeat variation) leads to rapid switching between RMSs with different methylation specificities, leading to differential regulation of genes that have particularly been shown to impact virulence (8).

The three Type I RMSs studied here, EcoUTI89I, EcoCFTI, and EcoKI, each methylate many hundreds (427–754) of distinct sites in their respective host genomes (and similar predicted numbers in other *E. coli* genomes). We tested for phenotypic impacts using an *in vivo* murine model of urinary tract infection, a complex phenotype which demands that inoculated cells react to changing nutrient, fluid flow, and host immune conditions. In addition, *E. coli* are known to occupy different niches within the urinary tract, ranging from bladder epithelial cells (both within the cytoplasm and on the surface) to the lumen of the bladder and the parenchyma of the kidney (102). While we did not investigate these different niches individually, there was no overall difference in infection titers, even during competition experiments with a wild type strain, a strong indication that no virulence defect is present when EcoUTI89I is deleted. Complementing this complex phenotype, we used the Biolog PM platform to screen a ‘simple’ growth phenotype in 1190 conditions, again finding no difference regardless of the presence or absence of the three Type I RMSs. Finally, we used RNA-seq to explore whether gene expression (either on a global scale or at the level of individual genes) was altered by changing methylation, again finding no differences attributable to DNA methylation. While RNA-seq was only performed under growth in LB, 60% of *E. coli* genes are transcribed under these conditions, and over 10,000 transcription start sites are used (69,103). Ultimately, however, it remains possible that under some specific conditions, these Type I systems would indeed alter gene expression or phenotypes; indeed, methyltransferases such as *E. coli* Dcm and *M. tuberculosis* MamA alter gene expression primarily under specific growth conditions such as stationary phase and hypoxia, respectively (77,91,104). (Notably, in the course of making one of the Type I RMS knockouts, we recovered a clone carrying a nonsynonymous mutation in a nucleotide biosynthesis operon; control experiments using this clone demonstrated that we could indeed detect differences in *in vivo* infections, PM arrays, and gene expression (manuscript in preparation)).

Alternatively, it may be reasonable that there is truly no regulatory impact of the Type I systems that we have studied (and, by extension, potentially other restriction systems). This alternative may not be so surprising, as Type I RMSs are the least common of the four classes of RMSs and have the longest recognition sequences, leading to fewer modified sites in a given genome (11,39,75). Indeed, the three Type I systems studied here each encode an order of magnitude fewer methylation sites than the known epigenetic regulator Dam (which methylates a short palindromic Type II motif). The native MG1655, UTI89, and CFT073 Type I systems are predicted to methylate ∼0.25–0.50% of promoters in the MG1655 genome (10–90 promoters out of up to 16,000 transcription start sites). Interestingly, while EcoKI and EcoUTI89I sites are underrepresented in intergenic sequences, EcoKI is not statistically underrepresented in the 50 bp upstream of the transcription start sites as predicted by any of these studies (68–70). For only one set of predicted TSSs (70), the EcoUTI89I site is significantly depleted (after correcting for multiple tests), while Dam sites (which are much more common and known to affect regulation) are significantly underrepresented in the sequences upstream of all sets of predicted TSSs (68,69) (Supplementary Table S8). Therefore, the lack of regulation by the Type I RMSs that we have observed may actually be due to chance (with a low number of sites, the probability of a methylation site being in the correct position to alter binding of RNA polymerase or any transcription factors is also lowered). Alternatively, it might be possible that the promoters or the restriction sites themselves (or both) have evolved to avoid any impact of methylation by these systems on regulation (which might be related to their common location in the Immigration Control Region (ICR), which is classically associated with restriction (87,105)), though we see no statistical evidence for this in our analysis.

In summary, we have demonstrated that, for 3 different strains of *E. coli*, the presence or absence of the native Type I RMSs have no measurable effect on gene expression and on any phenotype tested besides their originally described function of defense against incoming foreign DNA. This lack of regulation stands in contrast to other reports, thus suggesting fruitful avenues for future study. In particular, how general is the absence of regulation among *E. coli*, and is it limited to Type I RMSs? Do other bacteria also have RMSs, or orphan methyltransferases, that do not play an epigenetic role? Is the lack of regulation due to chance (with low numbers of recognition sites), or is there any adaptation of DNA binding proteins, promoters, or modification of RMS specificity? Future studies addressing these questions may provide further insights into the impact that RMSs (and, by implication, phages and other mechanisms that introduce foreign DNA) have had on bacterial genome evolution.

## DATA AVAILABILITY

The PacBio sequencing data is available in the GenBank Short Read Archive under BioProject PRJNA474982 (106). The RNA-seq datasets generated and analysed during the current study are available in the GenBank Short Read Archive under BioProject PRJNA675406 and GEO accession GSE165421.

## Supplementary Material

gkab530_Supplemental_Files
