# Supplementary material for: DNA methylation by three Type I restriction modification systems of Escherichia coli does not influence gene regulation of the host bacterium
Source: Nucleic Acids Res. 2021 Jun 28;49(13):7375–88. doi: 10.1093/nar/gkab530 (PMC8287963; doi:10.1093/nar/gkab530)
Supplement: gkab530_Supplemental_Files [file gkab530_supplemental_files.zip › Mehershahi and Chen TypeI RMS - Supplementary figures.pdf]

Supplementary material for:

DNA methylation by three Type I restriction modification systems of *Escherichia coli* does not influence gene regulation of the host bacterium

Kurosh S. Mehershahi and Swaine L. Chen

SUPPLEMENTARY TABLES

Table S1. List of strains, plasmids and primers. Primer sequences include Type I RMS methylation sites highlighted in bold and restriction enzyme cleavage sites underlined.
Table S2. RNA sequencing quality metrics.
Table S3. Methylated motifs identified by SMRT sequencing in *E. coli* UTI89 and UTI89*ΔhsdSMR*. Methylated motifs identified in the *E. coli* UTI89 and UTI89*ΔhsdSMR* genomes are listed, with modified bases underlined. DNA modifications: N6-methyladenine (6mA) and C5-methylcytosine (5mC). N=A, T, G or C and W=A or T.
Table S4. Logarithmic phase differentially expressed genes by RNA-seq. Genes with $\log_{10}$ FDR <=0.05 and $\log_2$ fold change =>1.5 are considered significant and highlighted in bold. The top 15 genes for each comparison are listed according to increasing $\log_{10}$ FDR values. *denotes deleted genes/alleles and polar effects.
Table S5. Stationary phase differentially expressed genes by RNA-seq. Genes with $\log_{10}$ FDR <=0.05 and $\log_2$ fold change =>1.5 are considered significant and highlighted in bold. The top 15 genes for each comparison are listed according to increasing $\log_{10}$ FDR values. *denotes deleted genes/alleles and polar effects.
Table S6. Phenotypic differences identified by PM due to altered *E. coli* Type I methylation. Type I methylation mutants are compared to corresponding wt strains and phenotypic differences listed in descending value of quality score. Phenotypes with quality score >150 are considered significant and highlighted in bold. * denotes resistance conferred by presence of kanamycin selection cassette at *hsdSMR* locus.
Table S7. Distribution of Type I methylation motifs in *E. coli* UTI89, MG1655 and CFT073 genomes. *UTI89 genome includes the 5,065,741bp chromosome and 114,230bp plasmid (pUTI89).
Table S8. Analysis of methylation site distribution in the MG1655 genome. Bootstrapping analysis with 10,000 replicates looking at the relative enrichment or depletion of EcoKI, EcoUTI89I, EcoCFTI, and Dam methylation sites upstream of transcription start sites in MG1655. P-value is uncorrected and based on bootstrapping (see methods). An uncorrected $P < 0.0018$ is considered significant (i.e. $P < 0.05$ after Bonferroni correction) and highlighted in bold font.

SUPPLEMENTARY FIGURES

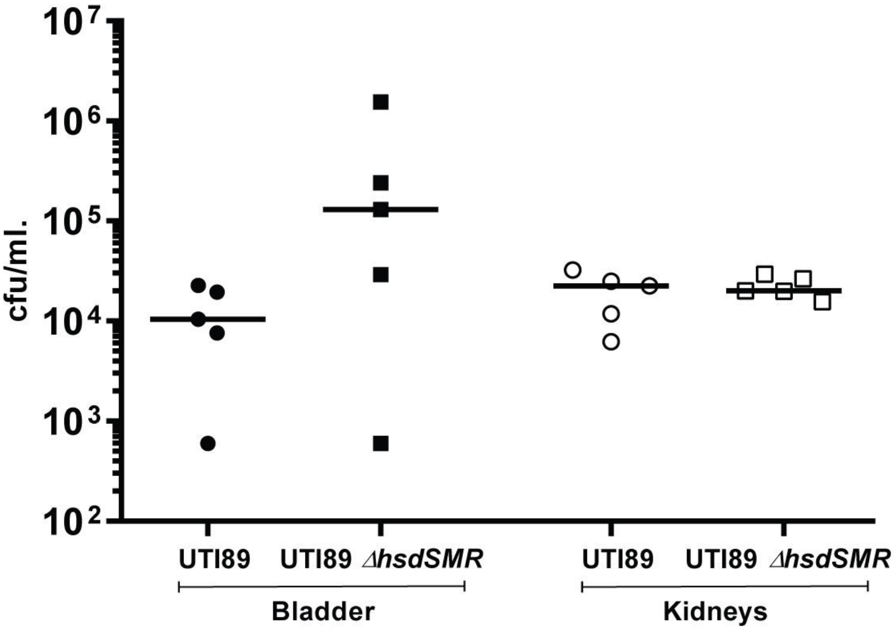

Fig S1. Loss of native Type I methylation does not alter UTI89 urovirulence. Wild type UTI89 or isogenic UTI89*ΔhsdSMR* strains were transurethrally inoculated (2×10^7 cfu/mouse). Bladder and kidney pairs (as indicated by the labels below the x-axis) were aseptically harvested at 1 day post-infection (dpi), homogenized and plated for determination of bacterial burden (colony forming units (cfu)/ml). Mann-Whitney test was used to identify significant differences between strains in bladders and kidneys; * $p < 0.05$, $n = 5$ mice/strain. Each data point represents a single mouse and horizontal lines represent the median values.

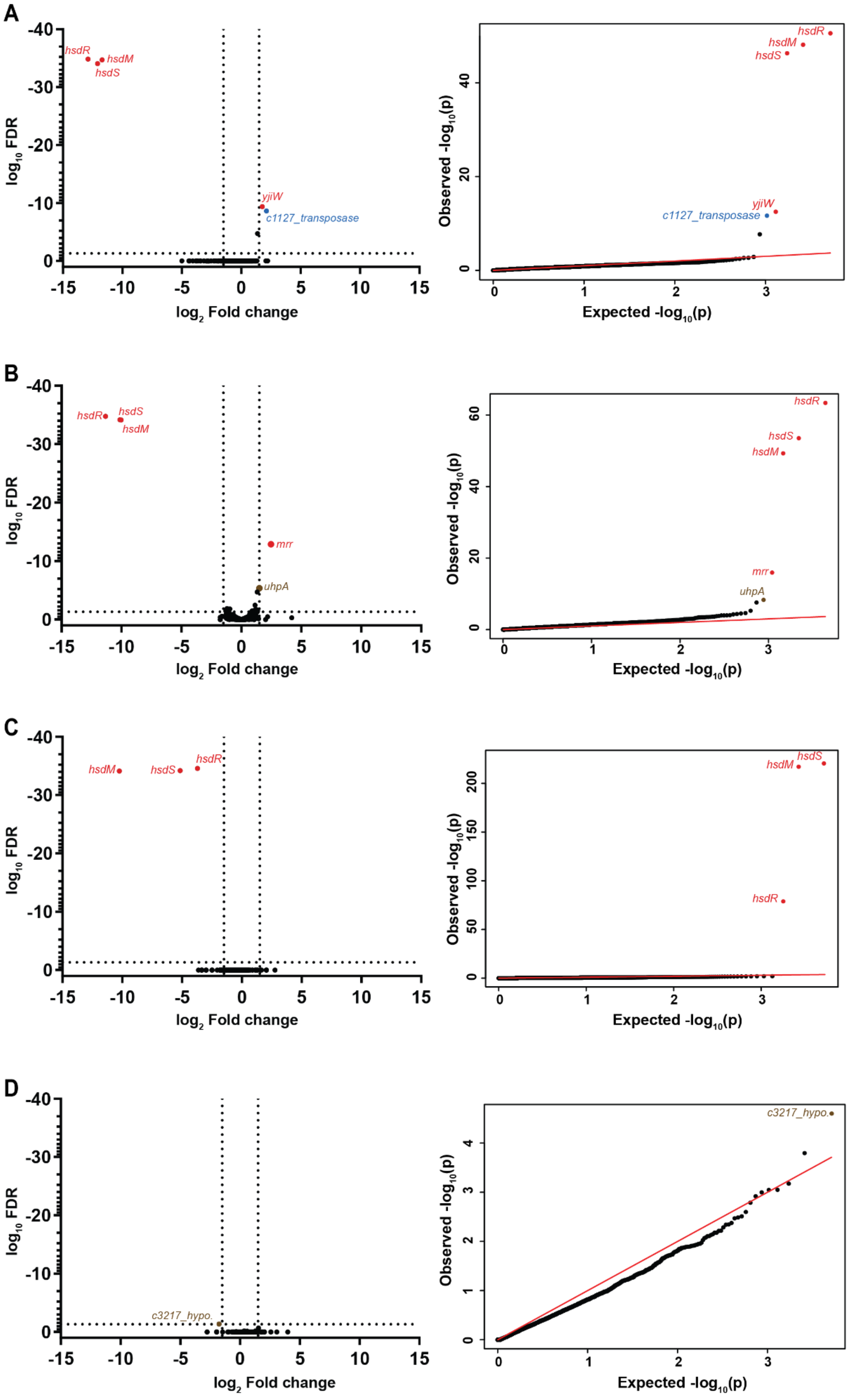
A
B
C
D
log10 FDR
log2 Fold change
Observed -log10(p)
Expected -log10(p)
hsdR
hsdM
hsdS
yjiW
c1127_transposase
mrr
uhpA
c3217_hypo.

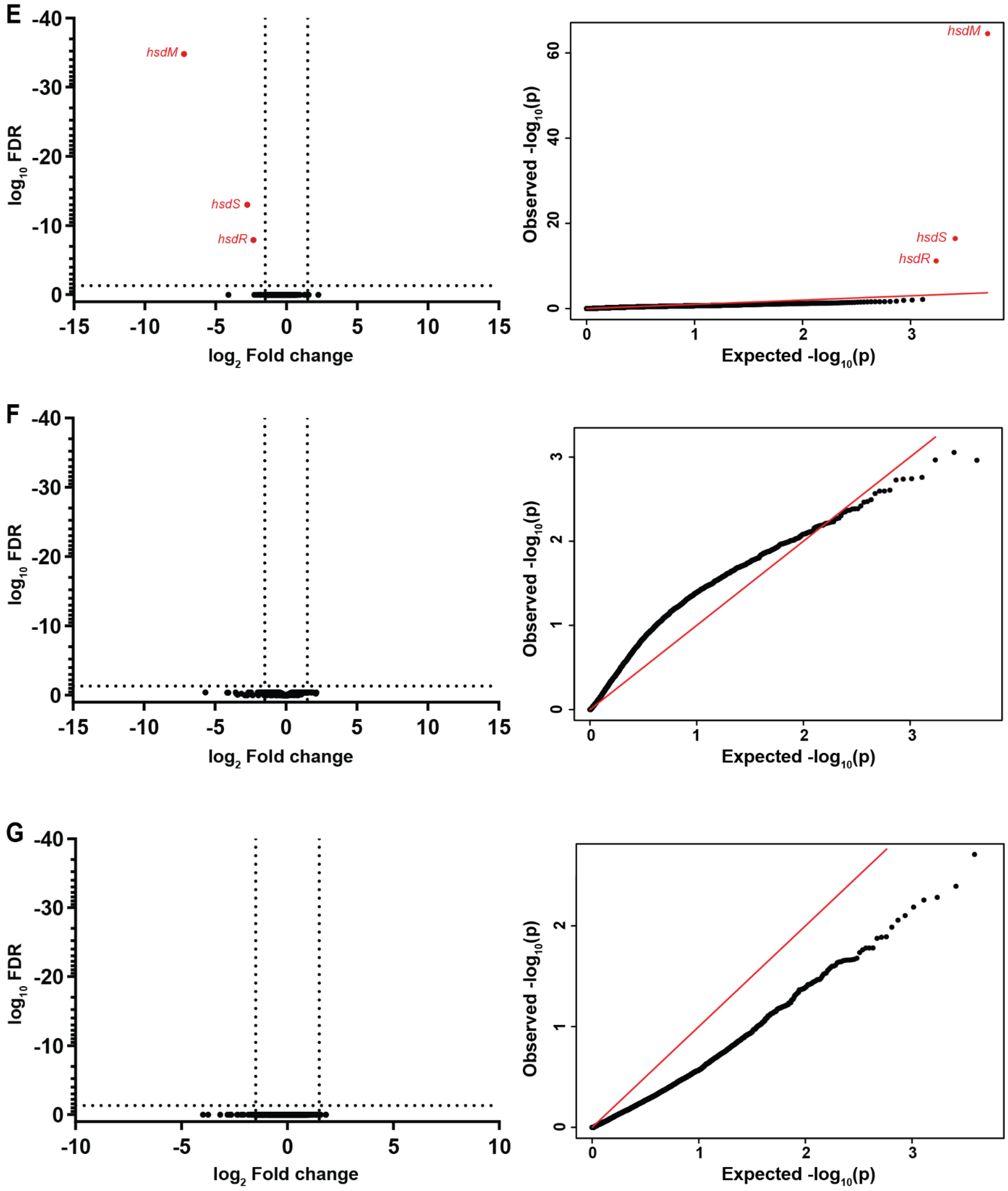

Fig S2. Perturbation of Type I RMS mediated methylation does not affect gene expression. RNA sequencing comparing transcriptomes of corresponding wt *E. coli* strain and **(A)** stationary phase UTI89*ΔhsdSMR,* **(B)** stationary phase MG1655*ΔhsdSMR*, **(C)** stationary phase CFT073*ΔhsdSMR*, **(D)** stationary phase UTI89 $hsdS^{MG1655}$, **(E)** stationary phase UTI89 $hsdSMR^{CFT073}$, **(F)** stationary phase UTI89 $hsdS^{UTI89}$, and **(G)** log phase UTI89 $hsdS^{UTI89}$. Left, a volcano plot of $\log_{10}$ FDR against $\log_2$ fold change. Right, qq-plots showing the distribution of uncorrected p-values. Significantly differentially expressed genes ($\log_2$ fold change >1.5 and $\log_{10}$ FDR <0.05) are labelled and colored either red (deleted genes/polar effects), brown (differentially expressed genes), or blue (validated as false positive by qRT-PCR). n = 3 biological replicates.

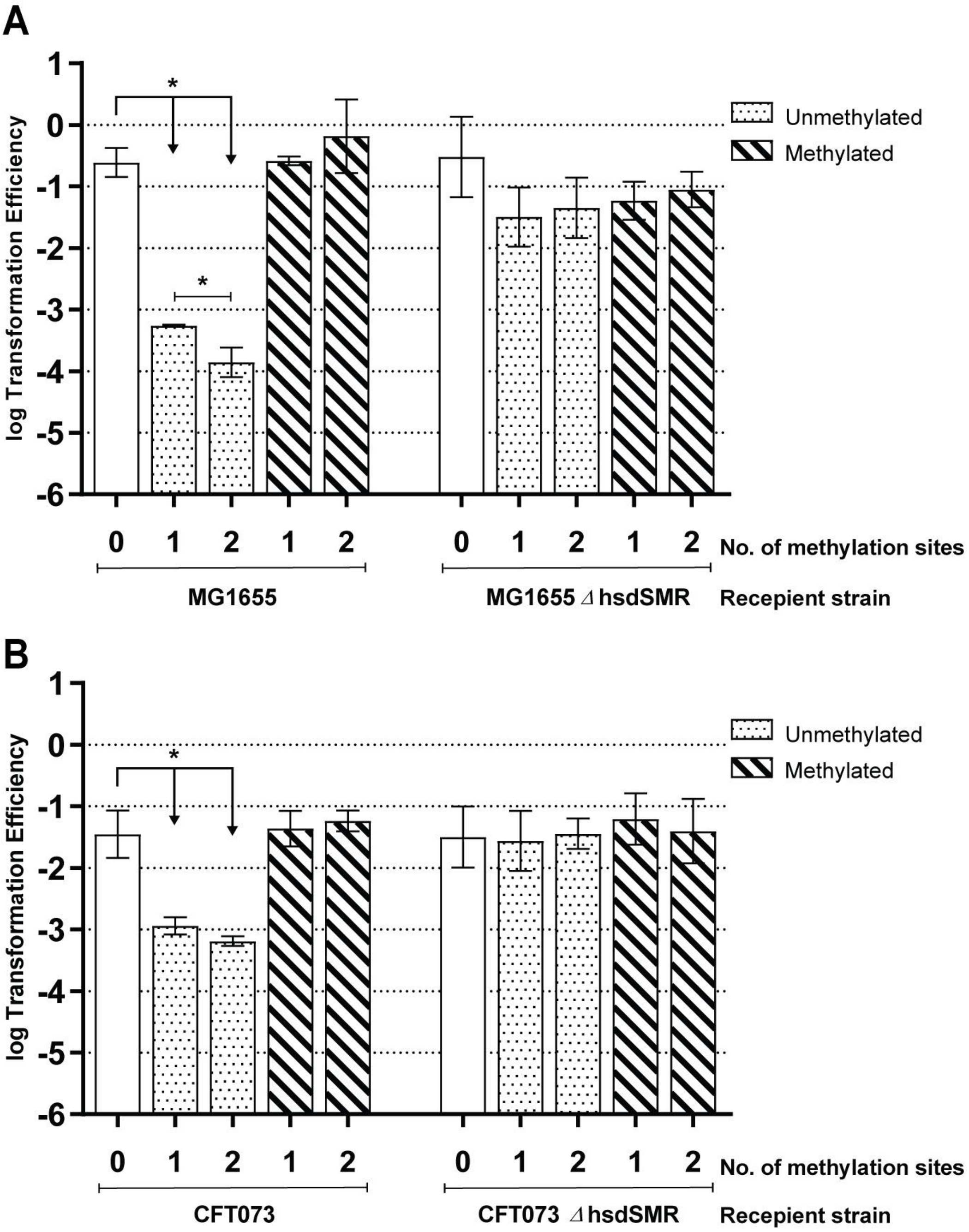

Fig S3. ***E. coli*** **strains MG1655 and CFT073 encode functional archetypal Type I RMSs with distinct specificities. (A)** Transformation efficiency assay using plasmids bearing 0, 1, or 2 copies (as indicated on the x-axis) of the MG1655 Type I RMS motif (5'-AAC(N_6)GTGC-3'). Recipient cells were wild type MG1655 and the isogenic *ΔhsdSMR* mutant, as indicated by the labels below the x-axis. **(B)** Transformation efficiency assay using plasmids bearing 0, 1, or 2 copies (as indicated on the x-axis) of the CFT073 Type I RMS motif (5'-GAG(N_7)GTCA-3'). Recipient cells were wild type CFT073 and the isogenic *ΔhsdSMR* mutant, as indicated by the labels below the x-axis. Unmethylated and methylated plasmid preparations were used to transform each strain, as indicated by the legend at the top right. An unpaired t-test was used to identify significant differences between plasmids with 0, 1 and 2 methylation sites for both preparations and strains; * $p < 0.05$, n = 3 biological replicates. Data represents mean ± s.d. of log transformed values.

A

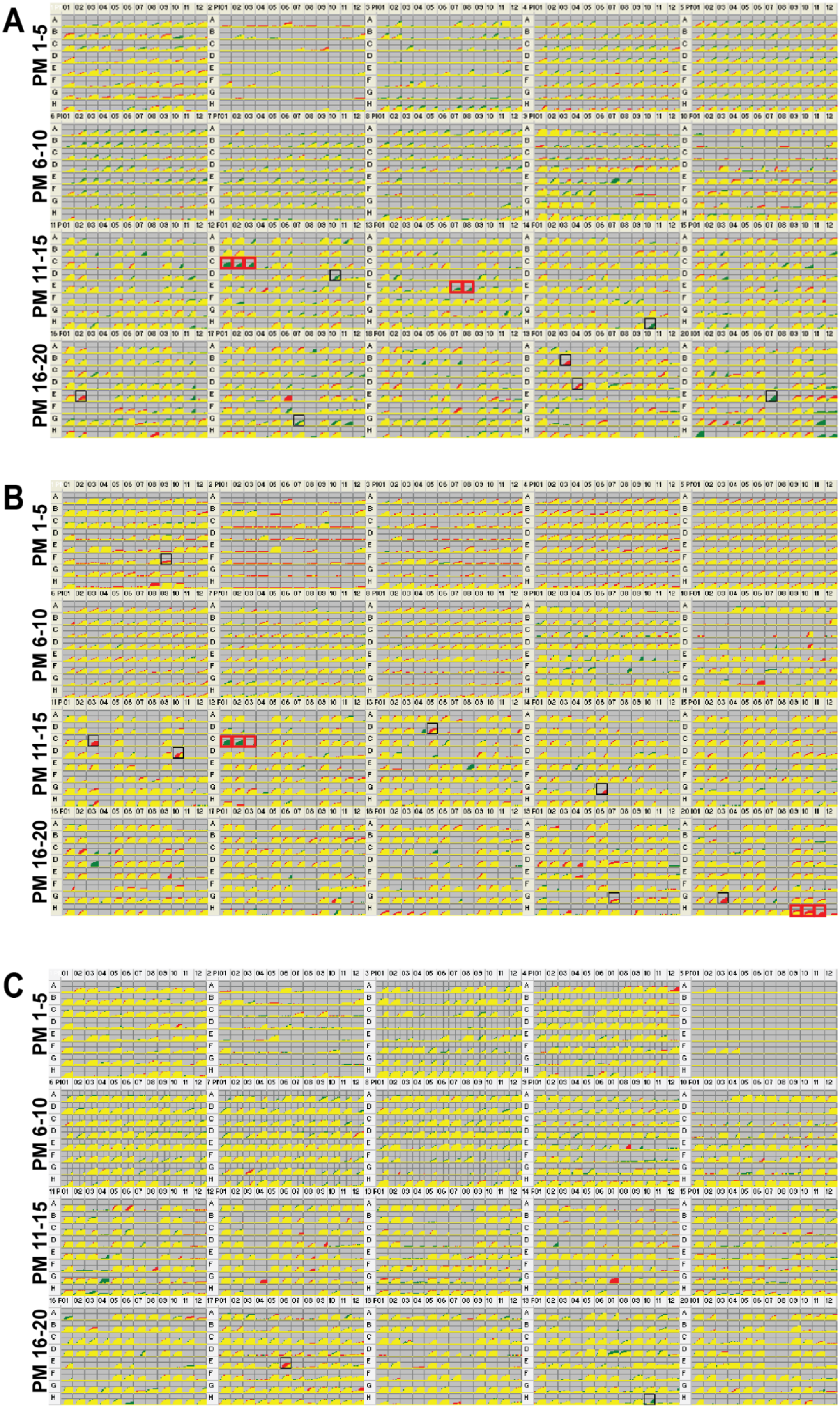

B

C

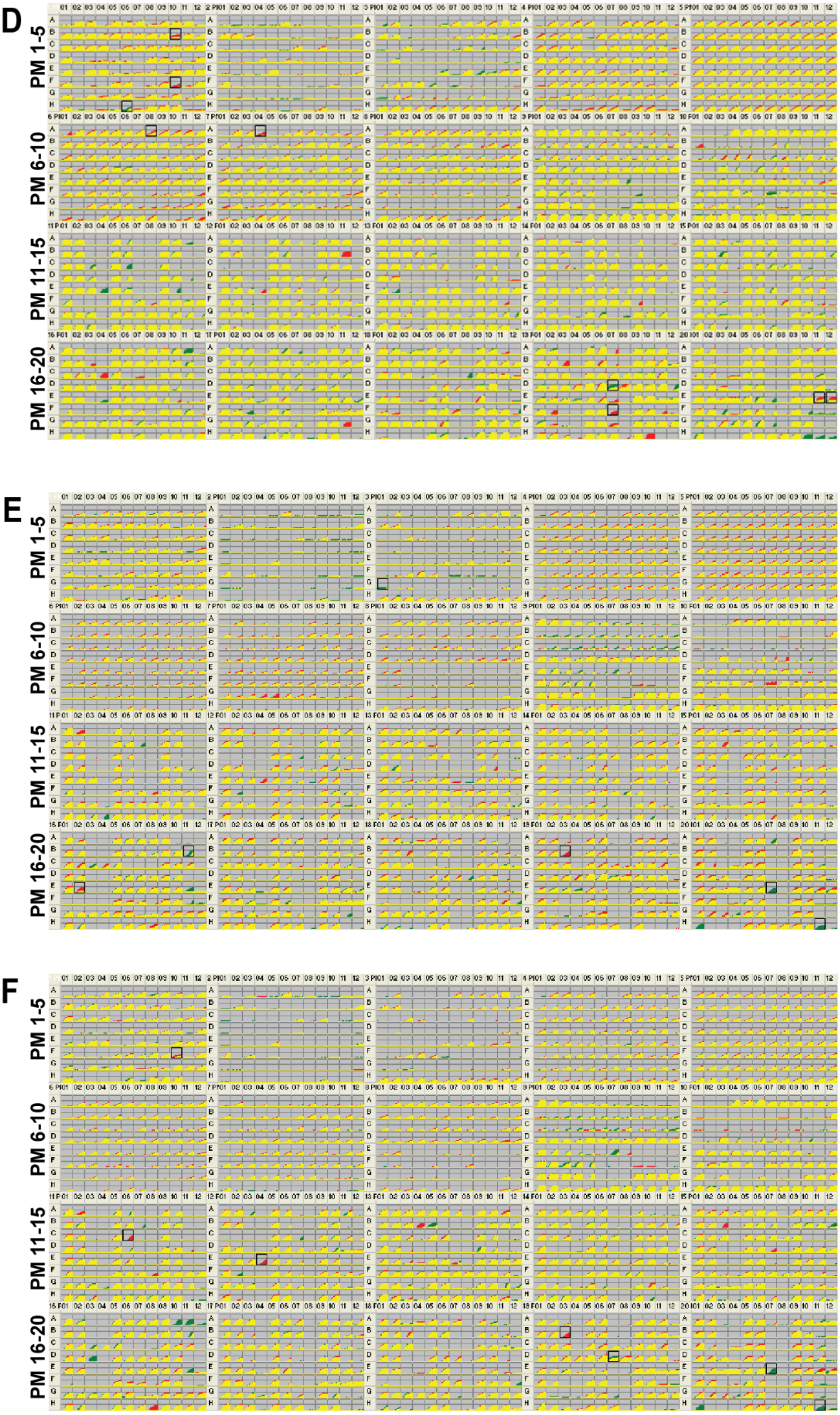
D
PM 1-5
PM 6-10
PM 11-15
PM 16-20
E
PM 1-5
PM 6-10
PM 11-15
PM 16-20
F
PM 1-5
PM 6-10
PM 11-15
PM 16-20

Fig S4. Altered Type I methylation has no effect on growth phenotypes. Phenotype microarray (PM) panel with plates PM1 to 20 comparing **(A)** wt MG1655 and MG1655*ΔhsdSMR*, **(B)** wt CFT073 and CFT073*ΔhsdSMR*, **(C)** wt UTI89 and UTI89 *hsdS*^{MG1655}, **(D)** wt UTI89 and UTI89 *hsdS*^{UTI89}, **(E)** wt MG1655 and MG1655 *hsdS*^{UTI89}, and **(F)** wt MG1655 and MG1655 *hsdS*^{MG1655}. Each plate is represented as a 12x8 grid of growth curves (red (wt), green (methylation mutant), and yellow (overlap) on a gray background). Each growth curve plots growth (measured colorimetrically) (y-axis) against time (x-axis). Wells representing conditions where a height difference was observed between the strains in both replicates are boxed in black, and wells which also have a quality score >150 are considered significant and boxed in red. n = 2 biological replicates.

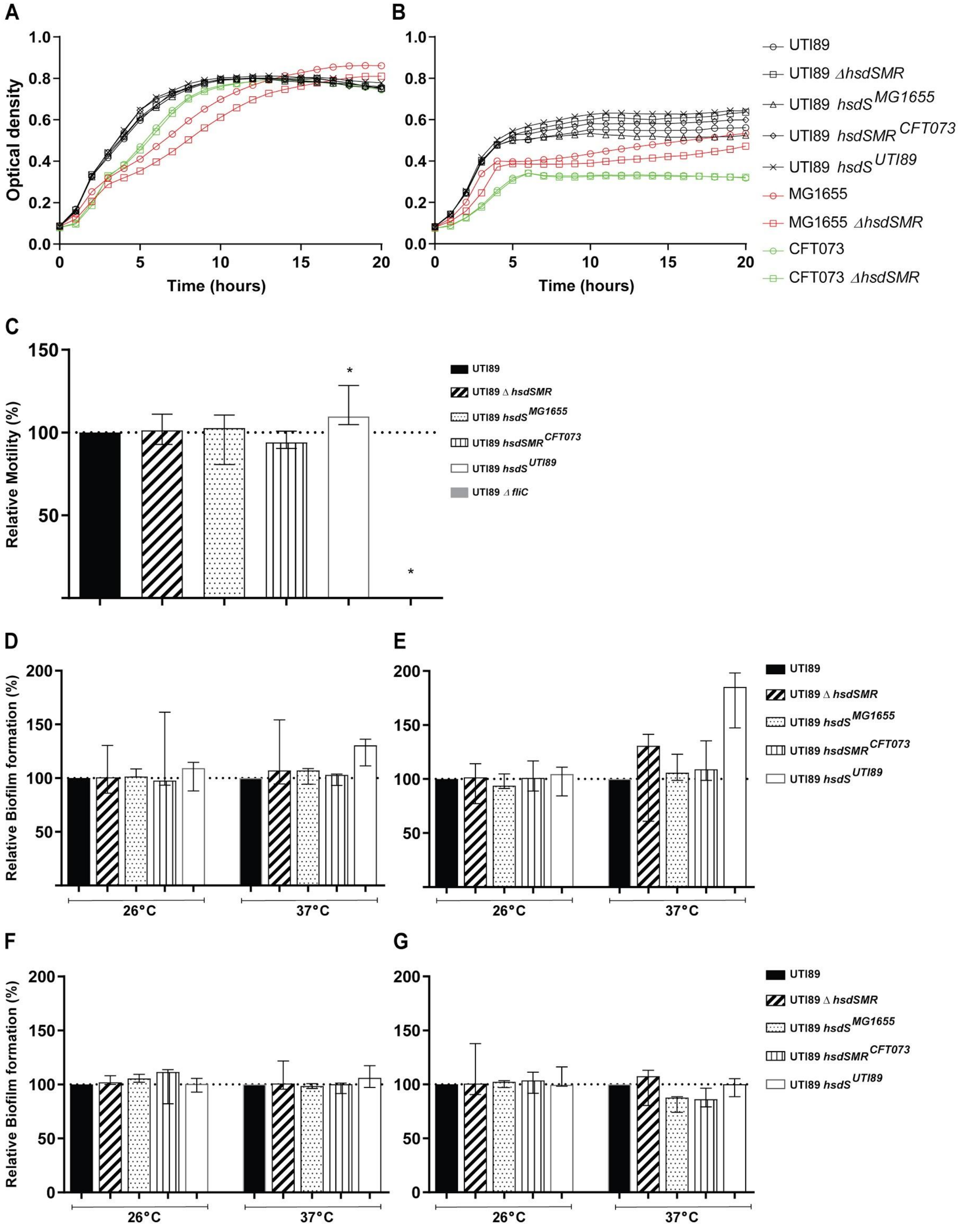

Fig S5. Type I methylation has no demonstrable effect using *in vitro* and *in vivo* virulence assays. (A and B) Growth curves generated using optical density (OD_{600}) values in **(A)** LB and **(B)** M9 minimal media. Wild type *E. coli* UTI89 (black), MG1655 (red), CFT073 (green), and corresponding Type I methylation mutant strains were tested, as indicated by the legend at the top right. Measurements were taken every 15 minutes and cultures propagated at 37℃. n = 3 biological replicates. Data points represent median values. **(C)** Soft agar assay used to measure motility of UTI89 methylation mutant strains, as indicated by the legend at the top right. Motility is represented relative to wild type UTI89 motility, which was included in each replicate. Strain UTI89*ΔfliC* lacking

flagellin serves as a non-motile control. Wilcoxon signed rank test was used to identify significant differences in bacterial motility relative to wild type; * $p < 0.05$, n = 3 biological replicates. Data represents median and 95% confidence intervals. **(D to G)** 96-well crystal violet assay for biofilm quantification using LB **(D and E)** or YESCA **(F and G)** media. UTI89 Type I methylation mutant strains were tested, as indicated by the legend at the top right. Assay was performed for 24 hours **(D and F)** or 48 hours **(E and G)**, as well as at 26℃ and 37℃ for each media as indicated by the labels below the x-axis. Biofilms were quantified using crystal violet absorbance at 590nm (OD_{590}) and represented relative to wild type UTI89 which was included in each replicate and condition. Wilcoxon signed rank test was used to identify significant differences in biofilm formation relative to wild type; * $p < 0.05$, n = 3 biological replicates. Data represents median and 95% confidence intervals.
